# Supplementary material for: Exosomal secreted SCIMP regulates communication between macrophages and neutrophils in pneumonia
Source: Nat Commun. 2024 Jan 23;15:691. doi: 10.1038/s41467-024-44714-4 (PMC10805922; doi:10.1038/s41467-024-44714-4)
Supplement: Supplementary file 1 — Supplementary information [file 41467_2024_44714_MOESM1_ESM.pdf]

Supplementary figures and tables

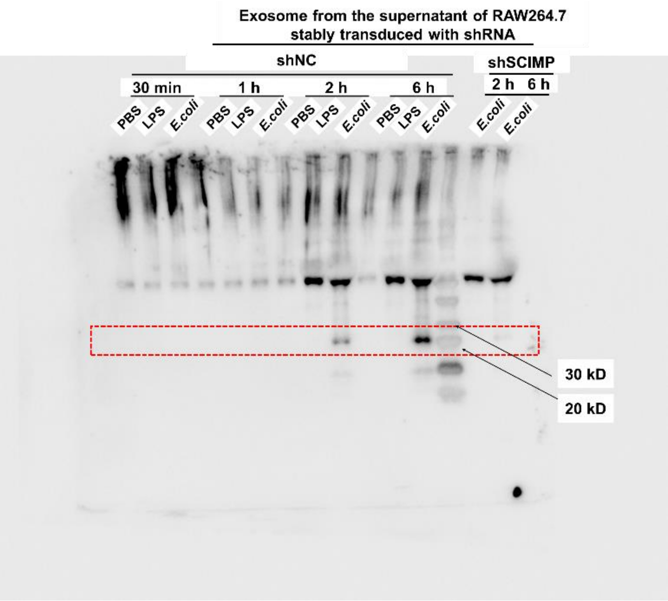

Figure S1 The shRNA of *Scimp* was designed and stably transduced into the RAW264.7 cells as well as the negative control shRNA. The two RAW264.7 cell lines were incubated with the PBS, LPS(1 μg/mL), or heat-denatured *E.coli* (MOI = 100) for 0.5, 1, 2, and 6 hours, and the exosomes in the supernatant were collected and purified. The endogenous SCIMP protein expression in the exosomes from the control RAW264.7 cells incubated with PBS, LPS, or heat-denatured *E.coli* for 0.5, 1, 2, and 6 hours and the Scimp knocked-down RAW264.7 cells incubated with heat-denatured *E.coli* for 2, and 6 hours, was detected by Western blot assay and stained with anti-SCIMP antibody. The sequence of shRNA to knock down the *Scimp* expression was "5'CCGGTCCGACAACCCTCAGCTTGGTACTCATTCAAGAGATGAGTACCAAGCTGAG GGTTGTC TTTTTG-3'" and the knocked down efficiency was evaluated by Q-PCR.

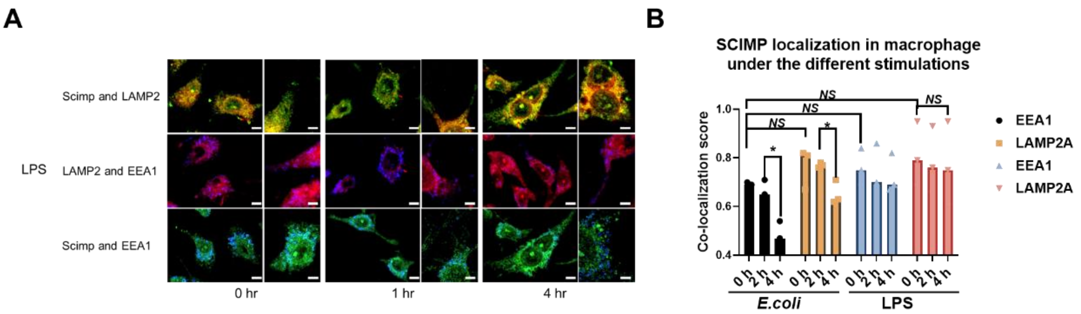

Figure S2. (A) After stimulation with LPS (1 μM) for 0, 1, and 4 hours, the RAW264.7 cells stably expressing SCIMP-GFP were fixed and permeabilized with Triton X-100 solution. Subsequently, LAMP2A and EEA1 were stained with fluorescein-conjugated antibodies (anti-LAMP2A: red; anti-EEA1: blue). The cellular localization of SCIMP-GFP, LAMP2A, and EEA1 was observed and recorded using a Dragonfly Confocal Microscope (scale bar = 100 μm). (B) Using the "Coloc2" algorithm in "ImageJ," the colocalization of SCIMP-GFP with EEA1/LAMP2A in the cells of each group (colocalization scores were obtained from more than ten cells in each group, n = 4) was calculated and statistically analyzed. Source data are provided as Source Data Supplementary Figures.

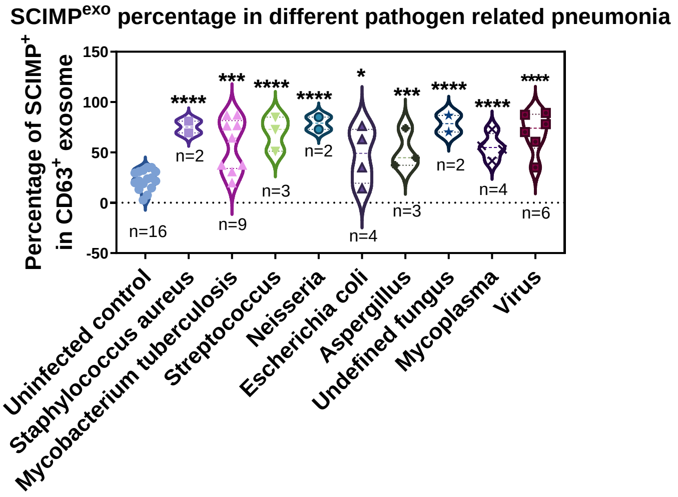

Figure S3. According to the infected pathogens type, the pneumonia cohort was divided into the bacteria subgroups, fungal subgroup, virus subgroup and mycoplasma subgroup, and the percentage of SCIMP$^+$ particles in CD63$^+$ particles in the BALF from all the pathogen subgroups was universally higher than in the pulmonary tumor cohort. Source data are provided as Source Data Supplementary Figures.

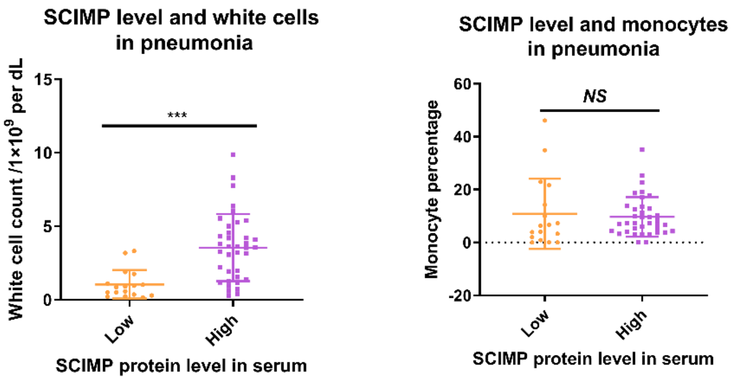

Figure S4. The whole white cells in the SCIMP protein level higher subgroup (>2000, n = 40 or <2000, n = 17) were much more than that in the SCIMP protein level lower subgroup, but not monocytes. Source data are provided as Source Data Supplementary Figures.

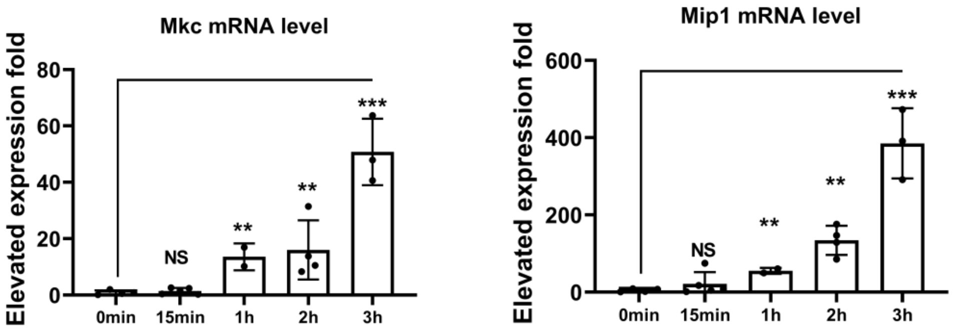

Figure S5. The classical chemokines, Mkc and Mip2, were observed began to be secreted into the BALF at about 1 hour in the *E.coli*-induced ALI model (n = 4 in each group). Source data are provided as Source Data Supplementary Figures.

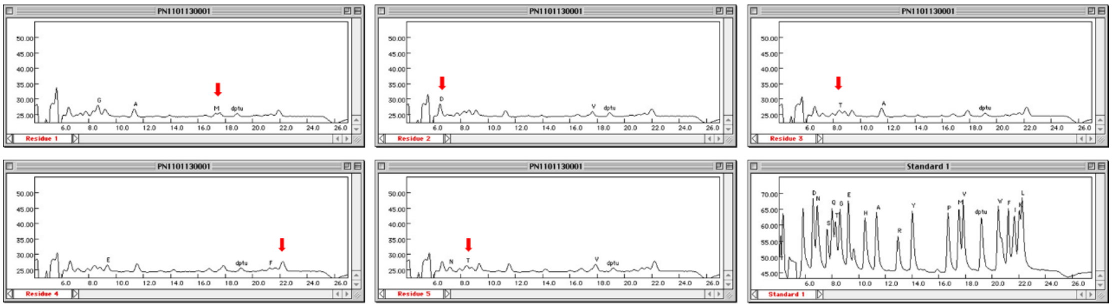

Figure S6. The amino acids in the mammalian expressed and purified SCIMP were measured by mass spectrometry and the first five amino acids in the sample were shown and labelled (red arrow, "MDTFTV").

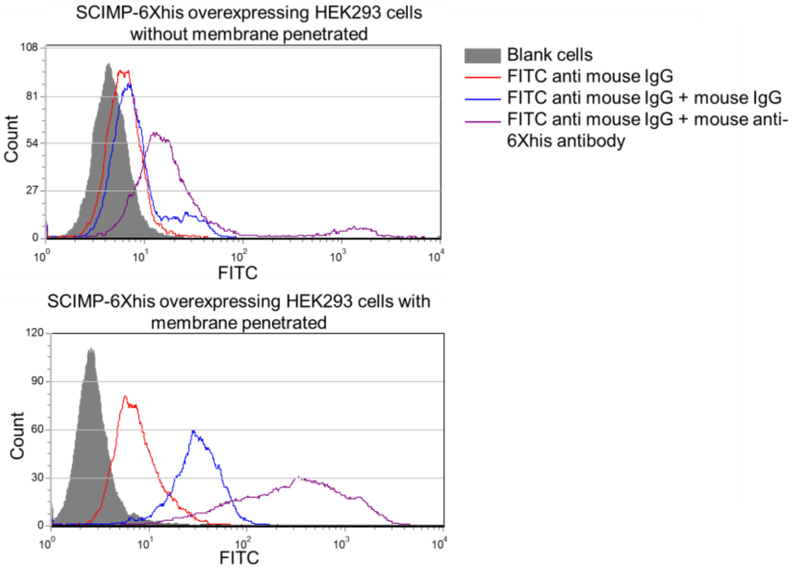

Figure S7. The HEK293 cells transiently transfected with SCIMP-6Xhis vector or the empty vector, which were fixed with or without membrane penetrated by Trixton-100, and the stained by the anti-6Xhis antibody or the isotype antibody and the fluorescence (FITC) of the cells was detected by flow cytometry.

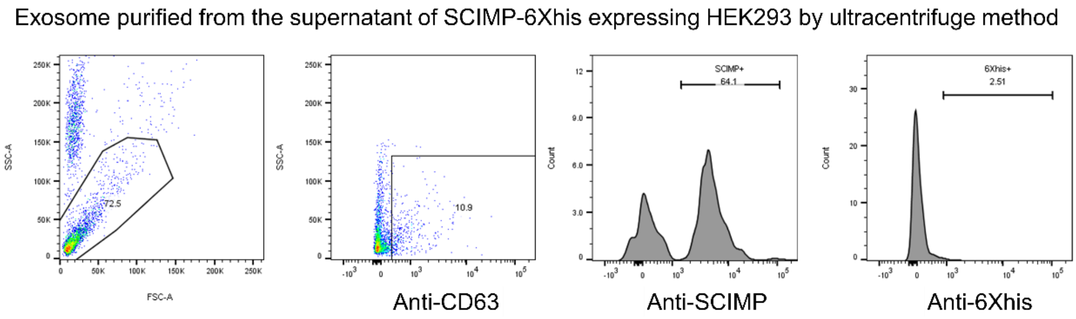

Figure S8. By using the FITC-SCIMP antibody and APC-anti-6×his antibody in the SCIMP^{exo} detection kit, we found the C-terminus of SCIMP was mainly at the inside of the exosomes purified from the supernatant of SCIMP-6×his overexpressing HEK293 cells.

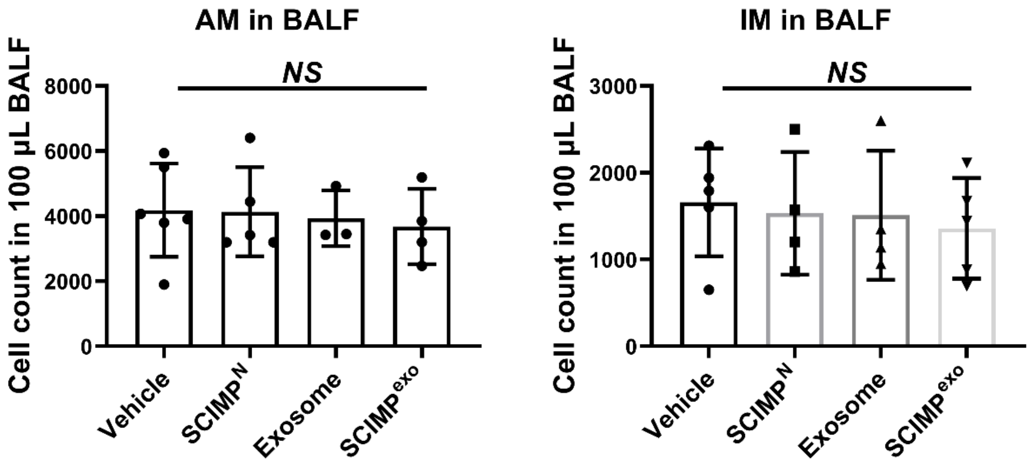

Figure S9. The cell counts of AMs and Infiltrating Macrophages (IMs) in the BALF at about 3-4 hours after the perfusion of SCIMP components to lung (n = 4 in each group). Source data are provided as Source Data Supplementary Figures.

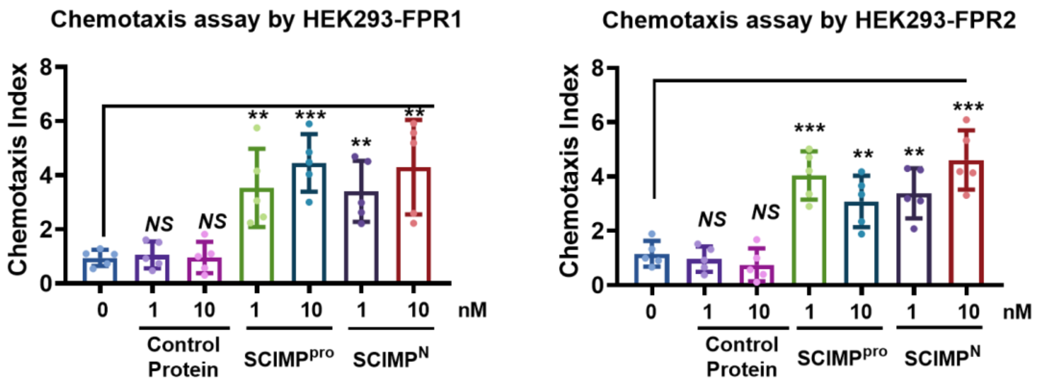

Figure S10. The chemotaxis activity of the control protein (prokaryotic PSMP, expressed and purified from bacteria), SCIMP protein (expressed and purified from bacteria) and SCIMP N terminus peptides (biosynthesized and purified by HPLC) to FPR1 or FPR2 overexpressing HEK293 cells in chemotaxis assay (Boyden chamber, chemoattracting time was 9 hours) with the concentration of 1, and 10 nM (n = 6 in each group). Source data are provided as Source Data Supplementary Figures.

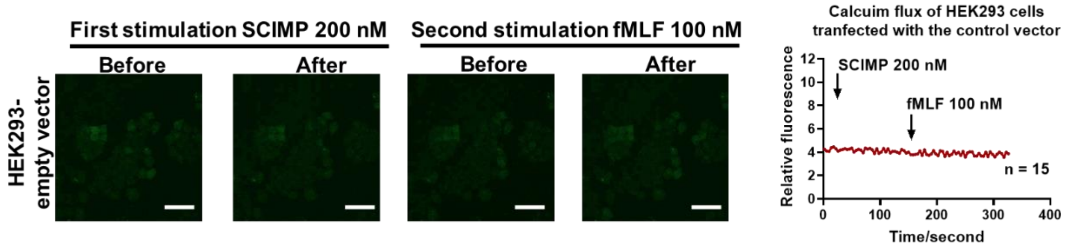

Figure S11. The calcium flux of the HEK293 cell transfected with the empty vector was detected by a time coursed confocal, and at the 20 second and 180 second of the observation the 200 nM SCIMP protein and 100 nM fMLF were added into the supernatant. Source data are provided as Source Data Supplementary Figures.

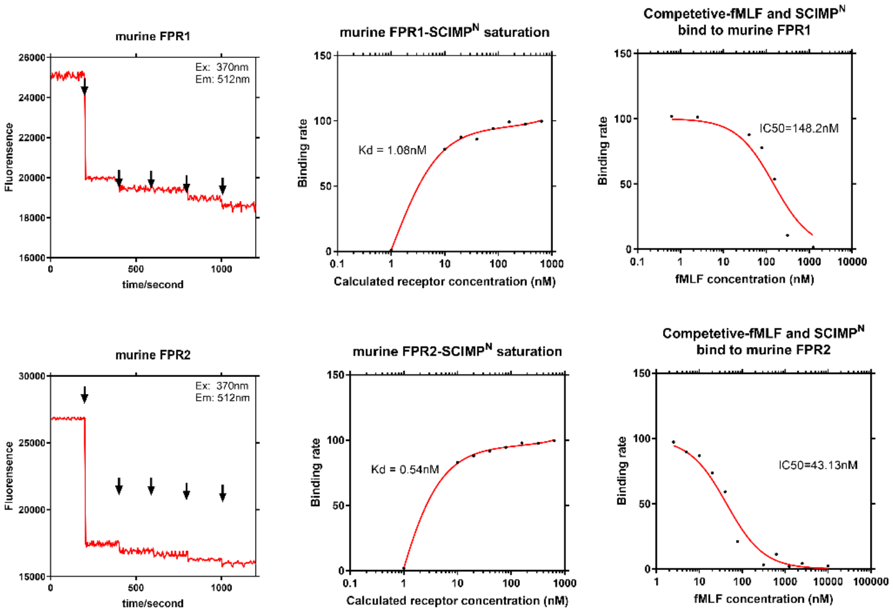

Figure S12. The murine SCIMP^N (final concentration was 1, 10, 20, 40, 80, 160, 320, 640 nM) were labeled by the dimer quenching probe, BODIPY FL Iodoacetamide, and then incubated with the fixed amount of membrane component of murine FPR1/2-HEK293 and the fluorescence density at the emission wavelength (512 nm) were measured, as well as the fMLF (5, 10, 20, 40, 80, 160, 320, 640 , 1280nM) to competitive binding to the murine SCIMP^N and murine FPR1/2. Source data are provided as Source Data Supplementary Figures.

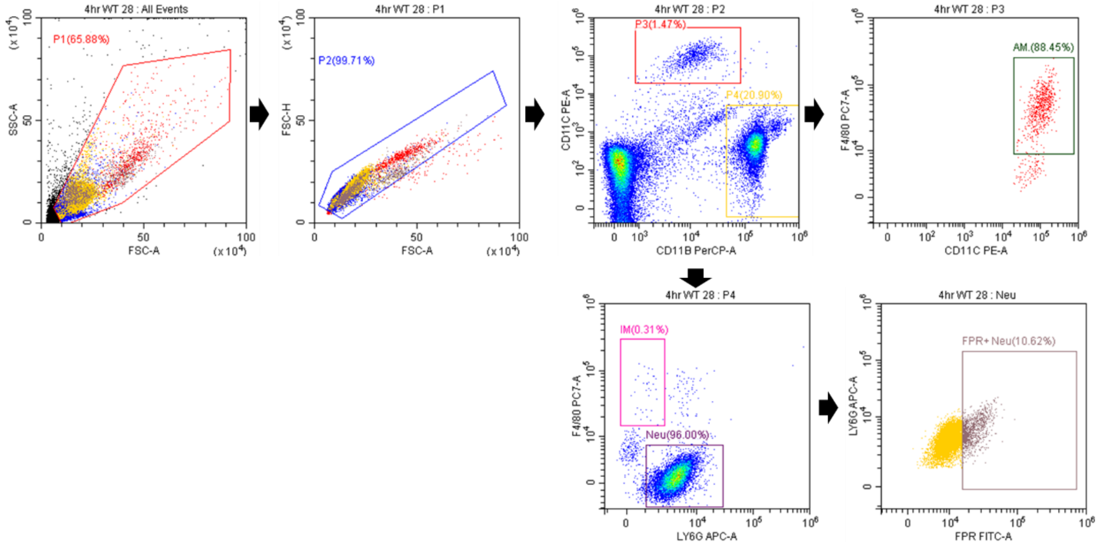

Figure S13. The gating strategy for neutrophils, alveolar macrophages, classical macrophages, and FPRs$^+$ neutrophils in the BALF, stained with antibodies and detected by flow cytometry, is presented.

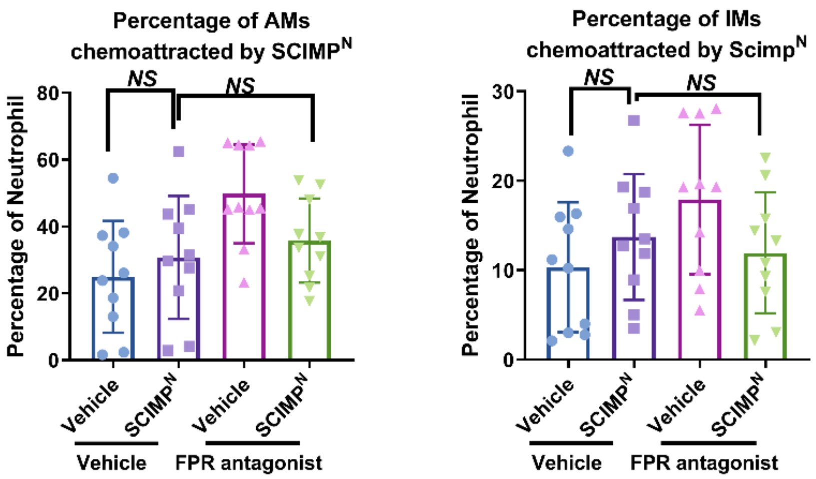

Figure S14. The chemotaxis of the Scimp N peptides and the vehicle (PBS) with or without the pretreatment of FPR antagonist (CsH, 50 μg per mouse) to the infiltrated classical macrophages (IMs, CD11b$^+$CD11c$^-$F4/80$^+$) or resident alveolar macrophages (AMs, CD11b$^+$CD11c$^+$F4/80$^+$) in the pulmonary of the C57 mice was measured by the flow-cytometry (n = 8 in each group). Source data are provided as Source Data Supplementary Figures.

Peptide1#: MDTFTVQDSTAMSWWRNN
Peptide2#: AKPLKHKQV
Peptide3#: NESPVQLPPLP
Peptide4#: QEAPSQPPATYSLVNKVK
Peptide5#: TVSIPSYI

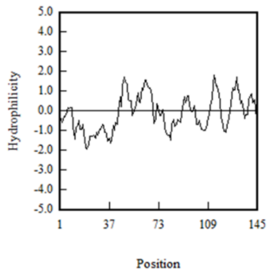

Figure S15. The peptides sequence from human SCIMP that were predicted to have high immune epitope, and “Peptide1#” is the N terminus of human SCIMP (left); The hydrophilicity of each amino acid in the human SCIMP protein were calculated (right).

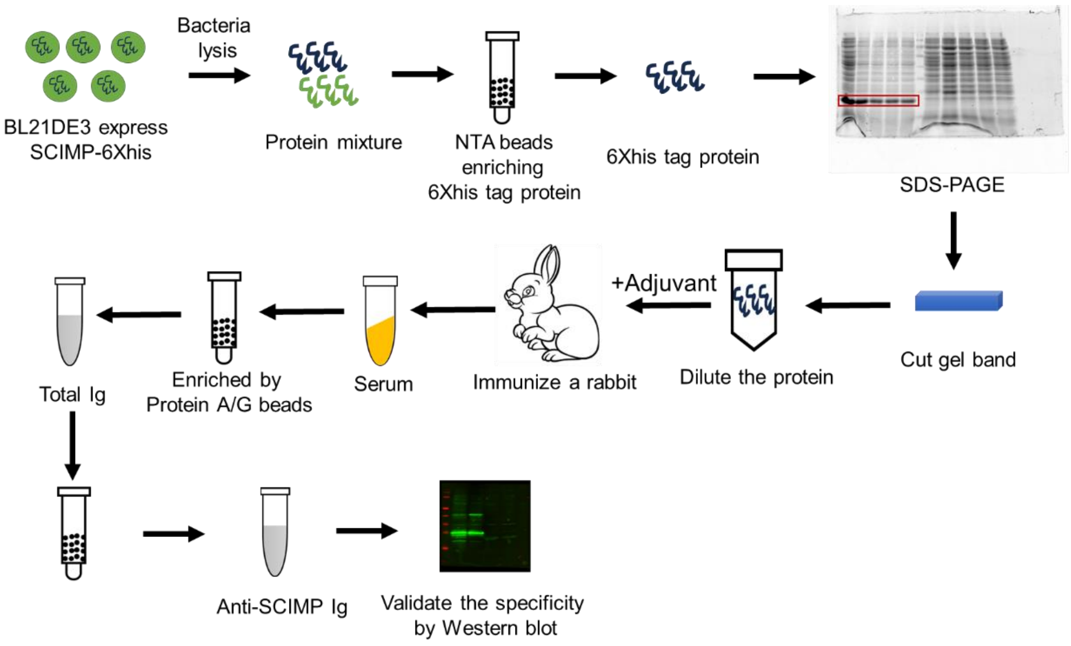

Figure S16. The processes to produce, purify, and evaluate the SCIMP antibody from immunized rabbit serum are schematically shown. The SCIMP antibody was generated by immunizing a rabbit with the purified prokaryotic SCIMP protein. After obtaining the SCIMP-immunized rabbit serum, Protein A/G-conjugated beads were used to extract the total antibody from the serum. Subsequently, the SCIMP-sourced peptides (listed in **Figure S14**) were conjugated to CNBr-activated Sepharose 4B and used to purify the antibody recognizing the peptides. The specificity of the SCIMP-specific antibody was evaluated by western blotting, demonstrating that the SCIMP antibody could recognize the exogenous SCIMP protein expressed by bacteria (BL21DE3) but not the lysate of bacteria with an empty vector transduced. The purified SCIMP antibody was then used in subsequent experiments.

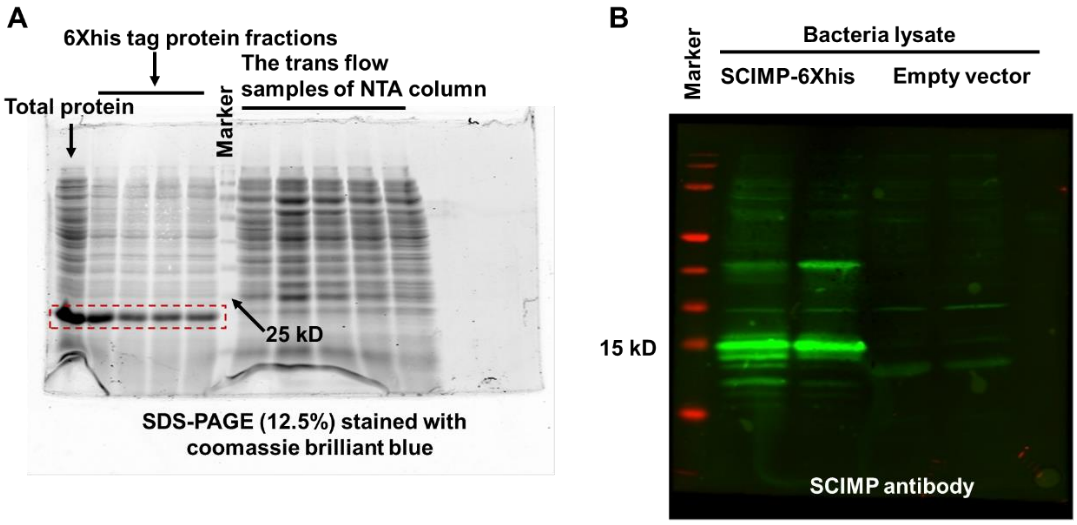

Figure S17. The human SCIMP-6×His recombinant protein was expressed in bacteria (BL21DE3), and the SCIMP-6×His protein in the bacterial lysate was purified using NTA Sepharose. The protein in each fraction during the purification process was analyzed by SDS-PAGE and stained with Coomassie blue (A). Additionally, the SCIMP-6×His protein in the bacterial lysate samples expressing SCIMP-6×His or transduced with an empty vector (pET32a) was probed with the purified SCIMP antibody in a Western blot (B).

Table S1. Basic clinical features of BALF samples providing patients

Clinical feature	infected cohort (N=36)	uninfected cohort (N=20)
Age (year), medium (range)	55 (29~78)	67 (37~78)
Gender, male perecentage (%)	23 (63.8%)	14 (70.0%)
Basic disease, count (%)		
No	15(41.7%)	15 (75%)
COPD	5(13.9%)	0
Pulmonary tuberculosis	2(5.6%)	0
Bronchiectasis	1(2.6%)	0
Post-transplant status of solid organs	4(11.1%)	0
SLE	2(5.6%)	0
DM	5(13.9%)	5(25%)
Nephrotic syndrome	2(5.6%)	0

* No: no basic disease, COPD: Chronic obstructive pulmonary disease, SLE: Systemic lupus erythematosus, DM: Dermatomyositis.

Table S2. Basic clinical features of serum samples providing patients

Clinical feature	Pneumonia cohort (N = 57)	Control Cohort (N = 50)
Age (year), medium (range)	33 (9~60)	37 (7~60)
Gender, male + female	32+25	30+20
Percentage (%)	(56.1%+43.9%)	(60.0%+40.0%)
Basic disease, count (%)		
AML	32(56.1%)	29(58.0%)
ALL	4(7.0%)	4(8.0%)
MDS	14(24.6%)	11(22.0%)
CML	1(1.8%)	1(2.0%)
SAA	5(8.8%)	4(8.0%)
PMF	1(1.8%)	1(2.0%)

*AML: Acute myeloid leukemia; ALL: Acute lymphoblastic leukemia; MDS: Myelodysplastic syndromes; CML: Chronic myeloid leukemia; SAA: Severe aplastic anemia; PMF: Primary myelofibrosis.
