# Supplementary material for: Exosomal secreted SCIMP regulates communication between macrophages and neutrophils in pneumonia
Source: Nat Commun. 2024 Jan 23;15:691. doi: 10.1038/s41467-024-44714-4 (PMC10805922; doi:10.1038/s41467-024-44714-4)
Supplement: Supplementary file 3 — Reporting Summary [file 41467_2024_44714_MOESM3_ESM.pdf]

nature portfolio

Corresponding author(s): Xiaolei Pei, Sizhou Feng, Ying Wang

Last updated by author(s): Dec 12, 2023

Reporting Summary

Nature Portfolio wishes to improve the reproducibility of the work that we publish. This form provides structure for consistency and transparency in reporting. For further information on Nature Portfolio policies, see our Editorial Policies and the Editorial Policy Checklist.

Statistics

For all statistical analyses, confirm that the following items are present in the figure legend, table legend, main text, or Methods section.

n/a	Confirmed	
☐	☒	The exact sample size (*n*) for each experimental group/condition, given as a discrete number and unit of measurement
☐	☒	A statement on whether measurements were taken from distinct samples or whether the same sample was measured repeatedly
☐	☒	The statistical test(s) used AND whether they are one- or two-sided<br>*Only common tests should be described solely by name; describe more complex techniques in the Methods section.*
☒	☐	A description of all covariates tested
☒	☐	A description of any assumptions or corrections, such as tests of normality and adjustment for multiple comparisons
☐	☒	A full description of the statistical parameters including central tendency (e.g. means) or other basic estimates (e.g. regression coefficient) AND variation (e.g. standard deviation) or associated estimates of uncertainty (e.g. confidence intervals)
☐	☒	For null hypothesis testing, the test statistic (e.g. *F*, *t*, *r*) with confidence intervals, effect sizes, degrees of freedom and *P* value noted<br>*Give P values as exact values whenever suitable.*
☒	☐	For Bayesian analysis, information on the choice of priors and Markov chain Monte Carlo settings
☒	☐	For hierarchical and complex designs, identification of the appropriate level for tests and full reporting of outcomes
☐	☒	Estimates of effect sizes (e.g. Cohen's *d*, Pearson's *r*), indicating how they were calculated

Our web collection on statistics for biologists contains articles on many of the points above.

Software and code

Policy information about availability of computer code

Data collection	Nanoflow&cytometry: CytoFlex S, BD Canto II;<br>TEM: Themis ETEM;<br>Confocoal: Andor Dragonfly Spinning Disk Confocal Microscope;<br>Chemotaxis: TaxiScan, Boyden Chamber;<br>Binding assay: Synergy H1<br>Mass spectrometry: Orbitrap Exploris 480
Data analysis	Statistics and graph: Graphpad prism9.0;<br>Image: FIJI imageJ;<br>Cytometry: FlowJo v10, Beckman Cytoexpert;<br>Proteomic: Proteome Discoverer 2.4, GOThe "coloc 2" packaged in the software "ImageJ" was used in this study to calculated the colocalization of the molecules in the cofocal pictures.<br>Function enrichment: GSEA, Go.

For manuscripts utilizing custom algorithms or software that are central to the research but not yet described in published literature, software must be made available to editors and reviewers. We strongly encourage code deposition in a community repository (e.g. GitHub). See the Nature Portfolio guidelines for submitting code & software for further information.

Data

Policy information about availability of data

All manuscripts must include a data availability statement. This statement should provide the following information, where applicable:

- Accession codes, unique identifiers, or web links for publicly available datasets
- A description of any restrictions on data availability
- For clinical datasets or third party data, please ensure that the statement adheres to our policy

The raw data used for graph and the original image of the Western blot in this paper are available as “Source Data Figures”. The raw data of the proteomic analysis are available as “Source Data Proteomic” and have been deposited in the ProteomeXchange (PXD046185, https://proteomecentral.proteomexchange.org/cgi/GetDataset?ID=PXD046185). The raw data used for the supplementary figures in this study are available as “Source Data Supplementary Figures”. If there are any questions on the data, method, study design or materials requirement, please contact peixiaolei@ihcams.ac.cn.

Research involving human participants, their data, or biological material

Policy information about studies with human participants or human data. See also policy information about sex, gender (identity/presentation), and sexual orientation and race, ethnicity and racism.

Reporting on sex and gender	The gender information of the participants is summarized in the supplementary Table.S1 and Table.S2
Reporting on race, ethnicity, or other socially relevant groupings	The race, ethnicity, or other socially relevant grouping is not used in this study.
Population characteristics	The population characteristics is summarized in the supplementary Table.S1 and Table.S2
Recruitment	During patient recruitment, participants were categorized into groups based on their clinical diagnoses: the pulmonary infection group, non-pulmonary infection group, and non-infection group. Among patients providing BALF samples, there were no differences in basic physiological indicators such as age, gender, and weight. However, the likelihood of comorbidities like COPD was higher in patients with lung infections. Patients contributing serum samples showed no disparities in age, gender, or weight, and they were initially diagnosed with blood system diseases, such as AML, MDS, ALL, without bias in the underlying disease types. After undergoing stem cell transplantation treatment for one month, sample collection commenced. The selection of pathogenic microbial types in all infected patients in this study was unbiased.
Ethics oversight	The Ethics Committee of Blood Diseases Hospital, Chinese Academy of Medical Sciences, and the Ethics Committee of Tianjin First Central Hospital.

Note that full information on the approval of the study protocol must also be provided in the manuscript.

Field-specific reporting

Please select the one below that is the best fit for your research. If you are not sure, read the appropriate sections before making your selection.

☒ Life sciences ☐ Behavioural & social sciences ☐ Ecological, evolutionary & environmental sciences

For a reference copy of the document with all sections, see nature.com/documents/nr-reporting-summary-flat.pdf

Life sciences study design

All studies must disclose on these points even when the disclosure is negative.

Sample size	The sample size was determined based on the results of previous trial experiments or experiments that had been conducted previously.
Data exclusions	Samples were excluded in cases where the cells count too less or singal too weak.Animals were excluded from experiments if they showed any signs of sickness (weight loss more than 20 %, skin infection, shaggy fur, loss ofor reduced movements, abnormal breathing).
Replication	Each experiment was successfully repeated at least three times under independent conditions.
Randomization	Our study subjects, apart from the observed characteristics, remained consistent in other aspects. They were randomly and equally divided into groups for respective treatments and observations.
Blinding	In experiments related to this study, the blinding approach is unnecessary, as it only involves variations in the treatment of the same experimental materials, such as cells or animals. The subsequent observations, measurements, and statistical analyses, which reveal whether our treatments are effective, do not require a blinding design.

Reporting for specific materials, systems and methods

We require information from authors about some types of materials, experimental systems and methods used in many studies. Here, indicate whether each material, system or method listed is relevant to your study. If you are not sure if a list item applies to your research, read the appropriate section before selecting a response.

Materials & experimental systems

n/a	Involved in the study
☐	☒ Antibodies
☐	☒ Eukaryotic cell lines
☒	☐ Palaeontology and archaeology
☐	☒ Animals and other organisms
☒	☐ Clinical data
☒	☐ Dual use research of concern
☒	☐ Plants

Methods

n/a	Involved in the study
☒	☐ ChIP-seq
☐	☒ Flow cytometry
☒	☐ MRI-based neuroimaging

Antibodies

Antibodies used	The antibodies, including anti-His antibody (MA1-21315-HRP, Invitrogen), anti-CD9 antibody (VJ1/20, Abnova), anti-CD63 antibody (353039, BioLegend), anti-CD81 antibody (abs132701, Absin), anti-TSG101 antibody (ab30871, Abcam), anti-EEA1 antibody (3288T, CST), anti-LAMP2A antibody (sc-20011, Santa Cruz), PerCP-Cy5.5-anti-CD11b antibody (101228, BioLegend), APC-anti-Ly6G antibody (127614, BioLegend), PE-anti-CD11c antibody (117308, BioLegend), PE-Cy7 anti-F4/80 antibody (123114, BioLegend), FITC-anti-FPR antibody (W15086B, BioLegend), and anti-CD9 capture beads (ab239685, Abcam), were commercially purchased.
Validation	The SCIMP antibody was generated by immunizing rabbits with purified prokaryotic SCIMP protein, and the purity of the SCIMP protein is shown in Fig.S14. After obtaining SCIMP-immunized rabbit serum, we evaluated the titer of the antibody in the serum that could recognize the SCIMP protein using directed binding ELISA. Protein A/G-conjugated beads were employed to extract the total antibody from the serum, and then the SCIMP-sourced peptides (listed in Fig.S14) were conjugated to CNBr-activated Sepharose 4B and used to accumulate peptides-recognizing antibody. The ability of purified antibody was assessed by recognizing the SCIMP-containing bacteria lysate or cell lysate (Fig.S16) and utilized in the subsequent experiments.

Eukaryotic cell lines

Policy information about cell lines and Sex and Gender in Research

Cell line source(s)	The HEK293, CHO, L929 and RAW264.7 cell lines were purchased from ATCC.
Authentication	No further authentication
Mycoplasma contamination	Negative
Commonly misidentified lines (See ICLAC register)	No commonly misidentified lines was used.

Animals and other research organisms

Policy information about studies involving animals; ARRIVE guidelines recommended for reporting animal research, and Sex and Gender in Research

Laboratory animals	C57BL/6J-SCIMPem1Smoc mice and C57BL/6J-Fpr1/2 deficient mice were generated by Shanghai Model Organisms Center, Inc.
Wild animals	C57BL/6 mice and WHBE rabbits were purchased from Beijing Vitalstar Biotechnology Co., Ltd.
Reporting on sex	The male and female mice were equally used in the animal models. The female rabbits were used in this study.
Field-collected samples	No field collected samples were used in the study.
Ethics oversight	All the procedures were approved and monitored by the Animal Care and Use Committee of the Institute of Hematology & Blood Diseases Hospital, CAMS & PUMC.

Note that full information on the approval of the study protocol must also be provided in the manuscript.

Flow Cytometry

Plots

Confirm that:

- [x] The axis labels state the marker and fluorochrome used (e.g. CD4-FITC).
- [ ] The axis scales are clearly visible. Include numbers along axes only for bottom left plot of group (a 'group' is an analysis of identical markers).
- [x] All plots are contour plots with outliers or pseudocolor plots.
- [x] A numerical value for number of cells or percentage (with statistics) is provided.

Methodology

Sample preparation	Cells in the BALF or PLF were labeled with FACS antibodies (PerCP- Cy5.5 CD11b, APC-Ly6G, PE-CD11c, PE-Cy7 F4/80, FPR1/FPR2 antibody [FITC], BV421-Ly6C).
Instrument	All the samples were analyzed by FACS Canto II and Cytoflex S.
Software	Flowjo v10 and CytExpert
Cell population abundance	The cells used for cytometry were no less than 1e5 per tube.
Gating strategy	The gating strategy in this study was shown in Figure S16.

- [x] Tick this box to confirm that a figure exemplifying the gating strategy is provided in the Supplementary Information.
